# Influence of SARS-CoV-2 variants and corticosteroid use on the effectiveness of baricitinib therapy in critical COVID-19

**DOI:** 10.1186/s13054-025-05367-x

**Published:** 2025-03-22

**Authors:** Seung-Hun You, Moon Seong Baek, Tae Wan Kim, Sun-Young Jung, Won-Young Kim

**Affiliations:** 1 Department of Global Innovative Drugs, The Graduate School of Chung-Ang University, Chung-Ang University, Seoul, Republic of Korea; 2 Division of Pulmonary and Critical Care Medicine, Department of Internal Medicine, Chung-Ang University Hospital, Chung-Ang University College of Medicine, Seoul, Republic of Korea; 3 College of Pharmacy, Chung-Ang University, Seoul, Republic of Korea

Dear Editor,

We sincerely appreciate the insightful comments provided by Li et al. [1] regarding our correspondence, “Baricitinib versus tocilizumab in mechanically ventilated patients with COVID-19: a nationwide cohort study” [2], recently published in *Critical Care*. We recognize the need for further clarification on certain aspects to ensure a more precise interpretation of our study findings.

We acknowledge that the emergence of multiple SARS-CoV-2 variants over time could have influenced the therapeutic efficacy of baricitinib and tocilizumab in critically ill patients with COVID-19. Our cohort analysis accounted for temporal variations by stratifying patients based on their inclusion earlier or later in the pandemic. To provide a more granular assessment, we reanalyzed the outcomes by stratifying them according to the predominant SARS-CoV-2 strains circulating in Korea during the study period: Delta (July 2021–December 2021) and Omicron (January 2022–October 2022). The wild-type and Alpha periods were excluded from the analysis, as baricitinib and tocilizumab had not yet been authorized for use in Korea during these phases. Our findings revealed no significant differences in outcomes between patients receiving baricitinib or tocilizumab during the Delta-dominant period. However, during the Omicron-dominant period, patients treated with baricitinib exhibited significantly lower 30-day mortality rates compared to those receiving tocilizumab (53.8% vs 61.5%; odds ratio 0.73; 95% confidence interval 0.56–0.95). This finding aligns with a previous cohort study demonstrating that baricitinib was more effective in mitigating the inflammatory response triggered by the Omicron variant [3]. Among the 98 of 1630 (6.0%) patients hospitalized due to reinfection, only 14 required mechanical ventilation (MV). Consequently, it was not feasible to determine whether the clinical outcomes associated with baricitinib differed in this subgroup of patients.

As noted by Li et al., the concurrent use of corticosteroids could potentially influence the immunomodulatory effects of baricitinib and tocilizumab. The authors suggested conducting a subgroup analysis based on corticosteroid use. However, nearly all patients (98.4% of the initial cohort) received corticosteroids. Therefore, instead of a binary analysis based on corticosteroid administration, we performed subgroup analyses of 30-day mortality based on corticosteroid dosage and treatment duration. Corticosteroid use was defined as the administration of at least one dose of intravenous dexamethasone, hydrocortisone, or methylprednisolone during hospitalization. To standardize comparisons, all doses were converted to prednisolone equivalents [4]. The cut-off values for daily steroid dose and total treatment duration were determined based on the mean values observed in the study population. Among patients who received corticosteroids for fewer than 12 days or at a daily dose below 60 mg, the baricitinib group exhibited significantly lower mortality rates than the tocilizumab group (Fig. 1). However, when corticosteroid treatment extended to 12 or more days, or when the daily dose reached 60 mg or higher, no significant differences in 30-day mortality were observed between the groups. These findings suggest that the beneficial effects of baricitinib may be more pronounced in patients with critical COVID-19 receiving lower corticosteroid doses. Further studies are warranted to investigate the interplay between corticosteroids, other immunomodulatory agents, and clinical outcomes.

**Fig. 1:**
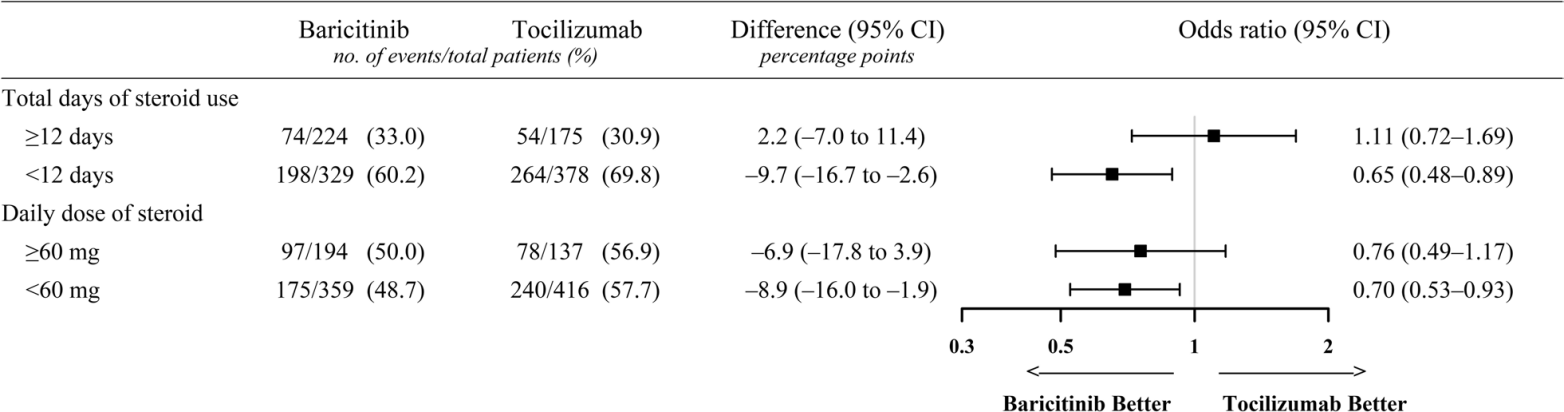
Association of baricitinib on 30-day mortality based on corticosteroid dosage and treatment duration. Odds ratios (represented by squares) and 95% CIs (depicted by horizontal lines) were calculated for the baricitinib (n = 553) and tocilizumab (n = 553) groups. *CI* confidence interval

Contrary to the authors’ comment, we had provided the duration of therapy with baricitinib and tocilizumab in the original paper [2]. In both the unmatched and propensity score-matched groups, the median (interquartile range) durations of baricitinib and tocilizumab use were 8 (4–13) days and 1 (1–1) day, respectively. The duration of baricitinib use aligns with previously reported values (7–8 days) [3, 5]. The daily dose of each drug was not calculated, as prescriptions may not always accurately reflect actual treatment. However, based on Korean COVID-19 treatment guidelines [6], the dosage was likely consistent at approximately 4 mg/day for baricitinib and 8 mg/kg/day for tocilizumab. We would like to clarify that baricitinib was associated with lower 30-day mortality than tocilizumab in the overall cohort of patients with COVID-19 requiring MV. This finding contrasts with previous studies that reported no significant benefit of baricitinib in mechanically ventilated patients [7, 8]. As noted by Li et al., the efficacy of baricitinib may be reduced in patients with gastrointestinal malabsorption or renal dysfunction, both of which are frequently observed in those receiving MV [8, 9]. However, the administration of baricitinib over multiple days may ensure more stable drug concentrations. Furthermore, its association with reduced 30-day mortality has been consistently observed in patients undergoing renal replacement therapy.

Among mechanically ventilated patients with COVID-19, baricitinib was associated with lower 30-day mortality than tocilizumab, particularly during the Omicron period and among those receiving lower doses of corticosteroids. We hope these clarifications address the concerns raised by the authors and further enhance the interpretation of our study findings.

## Data Availability

All data generated or analyzed during this study are included in this published article.

## Abbreviation

MV: Mechanical ventilation
